# Gaussian-Filtered High-Frequency-Feature Trained Optimized BiLSTM Network for Spoofed-Speech Classification

**DOI:** 10.3390/s23146637

**Published:** 2023-07-24

**Authors:** Hiren Mewada, Jawad F. Al-Asad, Faris A. Almalki, Adil H. Khan, Nouf Abdullah Almujally, Samir El-Nakla, Qamar Naith

**Affiliations:** 1 Electrical Engineering Department, Prince Mohammad bin Fahd University, P.O. Box 1664, Al Khobar 31952, Saudi Arabia; jalasad@pmu.edu.sa (J.F.A.-A.); akhan@pmu.edu.sa (A.H.K.); snakla@pmu.edu.sa (S.E.-N.); 2 Department of Computer Engineering, College of Computers and Information Technology, Taif University, P.O. Box 11099, Taif 21944, Saudi Arabia; m.faris@tu.edu.sa; 3 Department of Information Systems, College of Computer and Information Sciences, Princess Nourah bint Abdulrahman University, P.O. Box 84428, Riyadh 11671, Saudi Arabia; 4 Department of Software Engineering, College of Computer Science and Engineering, University of Jeddah, P.O. Box 34, Jeddah 21959, Saudi Arabia; qnaith@uj.edu.sa

**Keywords:** anti-spoofing, ASVspoof, convolutional neural network, genuine speech detection, voice conversion

## Abstract

Voice-controlled devices are in demand due to their hands-free controls. However, using voice-controlled devices in sensitive scenarios like smartphone applications and financial transactions requires protection against fraudulent attacks referred to as “speech spoofing”. The algorithms used in spoof attacks are practically unknown; hence, further analysis and development of spoof-detection models for improving spoof classification are required. A study of the spoofed-speech spectrum suggests that high-frequency features are able to discriminate genuine speech from spoofed speech well. Typically, linear or triangular filter banks are used to obtain high-frequency features. However, a Gaussian filter can extract more global information than a triangular filter. In addition, MFCC features are preferable among other speech features because of their lower covariance. Therefore, in this study, the use of a Gaussian filter is proposed for the extraction of inverted MFCC (iMFCC) features, providing high-frequency features. Complementary features are integrated with iMFCC to strengthen the features that aid in the discrimination of spoof speech. Deep learning has been proven to be efficient in classification applications, but the selection of its hyper-parameters and architecture is crucial and directly affects performance. Therefore, a Bayesian algorithm is used to optimize the BiLSTM network. Thus, in this study, we build a high-frequency-based optimized BiLSTM network to classify the spoofed-speech signal, and we present an extensive investigation using the ASVSpoof 2017 dataset. The optimized BiLSTM model is successfully trained with the least epoch and achieved a 99.58% validation accuracy. The proposed algorithm achieved a 6.58% EER on the evaluation dataset, with a relative improvement of 78% on a baseline spoof-identification system.

## 1. Introduction

Automation plays an essential role due to more responsive and efficient operations and tighter fraud-detection compliance. Automation saves time, effort, and money while decreasing manual errors and focusing on our primary goals. Automatic speaker authentication is a system that uses samples of human audio signals to recognize people. Entry controls to limited locations, access to confidentiality, and banking applications, including cash transfers, credit card authorizations, voice banking, and other transactions, can all benefit from speaker verification. With the increasing popularity of smartphones and voice-controlled intelligent devices, all of which contain a microphone, speaker authentication technology is expected to become even more prevalent in the future.

However, this technology’s vulnerability to manipulation of the voice using presentation attacks, also known as voice spoofing, poses a challenge. Various spoofs, such as speech synthesis (SS), voice conversion (VC), replay speech, and imitation, can be used to spoof automated voice-detection systems [1]. These possible attacks in speaker-based automation systems have been intensively examined in Reference [2], for example, microphone-based voice generation, feature extraction, and classifier- or decision-level attacks. In a replay attack, the perpetrator tries for physical access by playing a previously recorded speech that sounds like a registered speaker’s speech. The system is particularly vulnerable to replay attacks, as voices can easily be recorded in person or through a telephone conversation and then replayed to manipulate the system. Since replay attacks do not need a lot of training or equipment, these attacks are the most common and likely to happen. The ASVspoof 2017 dataset addresses the issue of replay spoofing detection. Previous works have extracted features that reflect the acoustic level difference between genuine and spoof speech for replay speech detection.

Mel frequency cepstral coefficients (MFCCs), linear prediction coefficients (LPCs), linear prediction cepstral coefficients (LPCCs), line spectral frequencies (LSFs), discrete wavelet transform (DWT) [3,4], and perceptual linear prediction (PLP) are speech feature extractions commonly used in speaker recognition as well as speaker spoofing identification [5]. A wavelet transform was used to obtain spectral features, and these features were integrated with CNN’s spatial features in Reference [6] for ECG classification. In Reference [7], the authors analyzed a 6–8 kHz high-frequency subband using CQCC features to investigate re-recording distortion. To record the distortions caused by the playback device, Singh et al. [8] derived the MFCC from the residual signal. A low-frequency frame-wise normalization in the (constant Q transform) CQT domain was suggested in Reference [9] to capture the playback speech artifacts. Deep feature-utilizing neural networks have also been studied for the recognition of playback speech in addition to these manually created elements. For instance, Siamese-embedded spectrogram and group delay were employed to teach deep features to the CNN [10]. However, feature extraction is highly dependent on DNN training, and it might be difficult to generalize it to ASV tasks that are performed outside of their intended domain.

**Figure 1:**
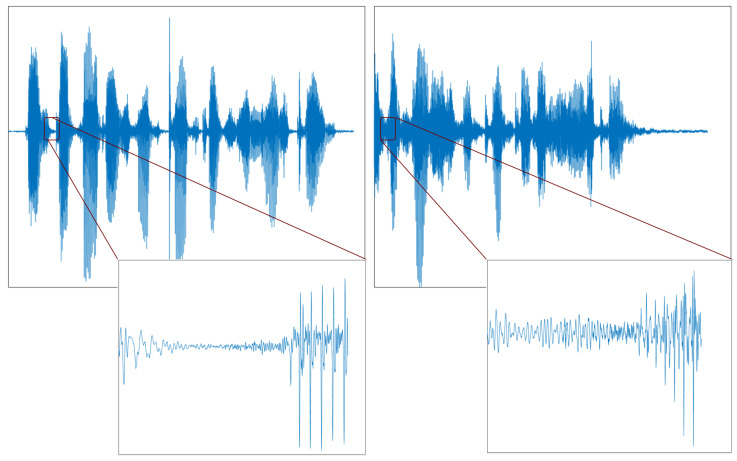
Time-domain speech signal of the same person’s (**upper**) genuine waveform and (**lower**) spoofed waveform.

The speech signal is processed in a ten-millisecond time frame without overlapping for future extraction from the speech. The speech signal is cleaved into two zones: the silent and speech zones. An area with low energy and an excessive zero-crossing rate is considered a silent zone, and an area with high energy is regarded as a speech zone. Huang and Pun [11] experimented with the same person’s genuine and spoofed speech signals using a replay attack. Figure 1 shows the actual and replayed speech signal, and it is observed that there is a difference in the silent segment shown in the red box. Thus, the use of a silent zone with a high-frequency region can discriminate the spoofed speech easily. A precise recording system is required for a replay attack. The background noise of a recording device is easily noticeable in a silent zone due to its low energy relative to a highly energized speech zone. However, finding a silent zone accurately is tricky. Therefore, the endpoint method of finding a zero-crossing rate and energy can be used to approximate the silent zone [12]. By adjusting the threshold of the zero-crossing rate detection and short-term energy, a speech and silent zone can be judged systematically.

Although the MFCC and CQCC are considered reliable features, the classifier’s performance can be significantly improved by combining them with complementary features, which can be done at the feature or score level [13]. Pitch, residual phase, and dialectical features are a few examples of complementary features. These complementary features, i.e., high pitch and corresponding phase, can easily be obtained at high frequencies [14].

The objective of this paper is to identify spoof speech against replayed attacks. This paper presents a new approach to concatenating high-frequency features with complementary features for better classification results. Generally, linear or triangular filter banks and their inversion are used to obtain high-frequency speech regions using filter banks. We emphasized the high-frequency features using a Gaussian filter in the proposed work.

One of the properties of the Gaussian filter is that it is a linear filter, which means that it preserves the correlation between different frequency components of the input signal. This is important in subband processing because it allows the different subbands to retain their correlation, which can be helpful in subsequent processing steps such as feature extraction or classification. The Gaussian function gives the frequency response of a Gaussian filter:

(1) $$H\left(f\right)=e^{-\frac{f^{2}}{2\sigma^{2}}}$$

where *f* is the frequency and σ is a parameter known as the standard deviation. This function has a bell-shaped curve centered at zero frequency and has a smooth transition to zero as the frequency increases. When the input signal is passed through a Gaussian filter, the filter response is applied uniformly across all frequencies, with higher frequencies being attenuated more than lower frequencies. This means that the different subbands created by the Gaussian filter will have a similar frequency response, with each subband containing a range of frequencies filtered by a bandpass filter but with the same Gaussian shape. This merit of the Gaussian filter can improve the accuracy of the processing result.

In summary, the following characteristic of Gaussian filters motivated us to use them for feature extraction: Firstly, the Gaussian filter allows for smooth switching between the subbands, maintaining the correlation between bands. Secondly, independent selection of the Gaussian filter’s mean and variance allows for controlled overlap between two consecutive subbands. Finally, the ease of filter design parameter calculation from the midpoint and endpoints of the original triangular filter bank is used in MFCC feature extraction. Nowadays, the convolution neural network (CNN) works amazingly in classification applications. However, the architecture and hyper-parameters of the CNN are critical because they control the training algorithm’s behavior, and it significantly impacts performance and training time. Therefore, the BiLSTM network, which considers the connection between the past and present features, was selected and optimized using the Bayesian algorithm. The optimization of BiLSTM gave minimal architecture with optimized hyper-parameters and helped with significantly improving the classification accuracy. The proposed model was validated with the well-known dataset ASVSpoof 2017. The key contributions are summarized as follows:

- A deep study on various feature sets used for classification is presented, and a multiple-feature integrated BiLSTM network is proposed.
- A conventional MFCC was obtained using a linear filter bank. We propose a new Gaussian-filtered inverted MFCC feature compared with conventional MFCC that provides a smooth transition between the subbands and maintains correlation within the same subband.
- RNN is the most effective method for spoof classification because it can handle short-term spectral features while responding to long-term temporal occurrences. LSTM networks overcome the vanishing gradients and long-term reliance problem of RNNs. BiLSTM was used in the proposed algorithm because the bidirectional strategy further improves recognition quality compared with the unidirectional approach.
- A Bayesian optimization algorithm, to optimize the hyper-parameters of BiLSTM while reducing its computational complexity and hidden layers, is presented.
- We used cutting-edge deep learning algorithms and compared their performance using assessment measurements.
- We present several issues based on the experimental evaluation and recommend possible solutions.

## 2. Related Work

Enormous studies on genuine and spoof speech signal classification have been proposed in past years. Major classification algorithms have two stages: a design of features extraction algorithm from the speech signal and a classifier to discriminate these features for speech classification. Many feature sets have been proposed with statistical and deep learning-based classifiers. A few widely used feature sets are as follows: Mel frequency cepstrum coefficients (MFCCs); inverse MFCCs (IMFCCs) [15]; linear frequency cepstrum coefficients (LFCCs); constant Q cepstrum coefficients (CQCCs) [16]; log-power spectrum using discrete Fourier transform (DFT) [17]; Gammatonegram, group delay over the frame, referred to as GD-gram [18]; modified group delay; All-Pole Group Delay [19]; Cochlear Filter Cepstral Coefficient—Instantaneous Frequency [20]; cepstrum coefficients using single-frequency filtering [21,22]; Zero-Time Windowing (ZTW) [23]; Mel-frequency cepstrum using ZTW [24]; and polyphase IIR filters [25]. The human ear uses Fourier transform magnitude and neglects the phase information [26]. Therefore, the phase spectrum has yet to gain attention in classification.

Along with features, the classifier also plays an important role. Many machine learning models have been proposed, including the Gaussian mixture model (GMM), K-nearest neighborhood (KNN), the hidden Markov model [27], support vector machine (SVM) [28], and convolution neural networks (CNNs). Multi-layer perceptron [29], deep CNN (DNN), and recurrent neural network (RNN) [30] are examples of widely used neural networks. The LSTM network is a type of RNN giving more memory power for an extended period, and it has been widely used in many applications. Ghosh et al. [31] used LSTM to remove the muscular artifacts from EEG signals. An energy-efficient speech recognition algorithm using LSTM was proposed in Reference [32]. This LSTM was implemented in CMOS, reducing energy requirements 2.19 times to the baseline model. The spikes’ temporal dependencies were captured from the EEG signals using LSTM for the brain–computer interface, which can help to evaluate emotion recognition [33].

In 2015, the first challenge, “Automatic Speaker Verification Spoofing and Countermeasures” [34], provided the dataset of spoofed speech signals based on synthetic speech, voice conversion, and other unknown attacks. The base algorithm using CQCC features and GMM as a classifier was presented with 24.77% EER. In this challenge, CQCC-based features showed promising results with an Equal Error Rate (EER) of 0.255% in Reference [35]. However, this ASPSpoof 2015 dataset does not contain replay attacks. Therefore, the dataset was revised, and the new dataset of ASVSpoof 2017 [2] was published, focusing on replay attacks. Again, using CQCC features and GMM as a classifier, the base algorithm secured a 24.77% EER, where GMM was trained using training and development datasets.

Xue et al. [36] presented a fusion approach using facial and speech features using convolution neural networks. The results were tested on ASVSpoof 2019 datasets, achieving a 9% EER rate. In Reference [37], the authors observed that the block-based approach missed the instantaneous spectral features. Therefore, single-frequency filtering was proposed, presenting high spectral and temporal resolution. Their model performed well, with a 0.05% EER on BTAS test data. A similar approach was presented in Reference [38], where instantaneous frequency and instantaneous energies were obtained using Hilbert transform, and genuine speech was differentiated from spoofed speech using empirical mode-decomposition features. They integrated these features with CQCC and group delay to improve performance. Their work also focused on replay attacks only. The voice quality features were combined with CQCC features to identify the replay attacks in speech signals in Reference [39]. Their work is limited to binary classification with replay attacks only. Chaudhari et al. [40] discussed three features, including LPC, CQCC, and MFCC, with GMM classifiers. They showed that combining MFCC and CQCC features enhanced the performance with a 10.18% EER. Glottal flow and an acoustic-based total of 106 features obtained from the speech signals were used in SVM and XGBoost classifier in Reference [41]. The XGBoost outperformed the SVM, resulting in a 98.8% classification accuracy. However, this model used extensive feature sets in the classification. Compatibility testing among a large number of devices is also challenging. Naith [42] conducted a test for Android and IoS devices. A total of 42 speakers participated in the creation of 219 datasets, a good and sufficient participation number for such empirical studies.

The integration of the well-established speaker modeling model “i-vector space” and the synthesis-channel subspace model was proposed with two-stage probabilistic linear discriminant analysis [43]. However, they tested the model with two voice-conversion attacks only. A capsule network is modified by replacing the ReLU with a leaky ReLU layer and a modified routing algorithm for better attention to the speech artifacts [44]. They focused on text-to-speech-based attacks in spoofing. The authors in Reference [45] extracted features using two partitioned datasets in logical and physical access. Later, they assembled the features by normalizing them and trained the CNN model by evaluating the loss function.

In Reference [46], cepstral features were obtained using single-frequency filtering. GMM and deep learning classifier models were compared. Later, a score-fusion approach was employed to improve the performance of the model by 17.82% EER in the evaluation dataset. Zhang et al. [30] employed a CNN and recurrent neural network (RNN) simultaneously. They trained this network using perceptual minimum variance distortionless response (PMVDR), teager energy operator-based critical auto-correlation envelope (TEO), and a spectrogram separately. They observed that spectrum-based features worked well with their network on ASVSpoof 2015 datasets, with an average EER of 0.36% compared with PMVDR and TEO, with EERs of 1.44% and 2.31%. Patil et al. [47] improved the potential of TEO using the signal mass in the front stage, and different classifiers, including GMM and light-CNN trained with 20 epochs, were tested in the second stage with ASVSpoof 2017 datasets. The GMM model performed well, with EERs of 5.55% and 10.75% on the development and evaluation datasets, respectively. In Reference [48], a group delay concatenated over the consecutive frames of the speech signal was used as a feature in the ResNET18 classifier. It showed a remarkable improvement, with zero EER on the development and evaluation datasets ASVSpoof 2017. However, the authors tested the model on a subset of the dataset, and the model’s validation for different types of attacks was not presented in the paper. Various extensions of ResNET using the Squeeze Excitation Network, including SENET34, SENET50, Mean-Std ResNET, and Dialted ResNET, proposed using CQCC features sets by Lai et al. [49]. The EER rate was reduced to 0.59 for the physical access dataset and to 6.70 for the logical access dataset of ASVSPoof 2019. They observed that further meta-data analysis and refinement in the algorithm is required.

Analysis of the deep RNN network was presented by Scardapane et al. [50]. They evaluated four architectures with MFCC features, log-filter bank features, and a concatenation of these two feature sets using ASVSpoof 2015 datasets. They observed that three LSTM layers trained with MFCC features gave better EERs than a log-filter bank. In contrast, a network combining three dense layers and three LSTM layers with MFCC features performed well, with 2.91% EER. Mittal and Dua [51] presented a hybrid deep CNN using static and dynamic CQCC features sets. Hybrid CNN combined the CNN-LSTM model with a time-distributed wrapper integrated into the LSTM network. This hybrid approach achieved a 0.029% EER on the evaluation dataset with high computation power. A standard time-delayed CNN (TD-CNN) was modified with a statistical pooling operation instead of max pooling, and angular softmax was used in the architecture in Reference [1]. The training of the TD-CNN model using third- and fourth-order moments achieved a 3.05% EER.

Dinkel et al. [52] tried to remove the crucial feature extraction step. First, they used the row form of speech frames as an input to the LSTM model to obtain features in the form of likelihood, and later, CNN was used for classification. However, no validation for unknown attacks was presented. Mittal and Dua [53] converted the CQCC features in 3D-tensor into 2D space, and a 2D-CNN was used for classification. A 3D tensor was obtained by reshaping the 30 statics and first- and second-order CQCC features. An RNN network was trained with cross-entropy and KL divergence loss for audio spoof classification in Reference [54]. Three variants of RNN were proposed in Reference [55]. MFCC, CQCC, and log-magnitude STFT features were used in the RNN, and they obtained a 25% improvement compared with the base model of GMM.

A light-CNN has been proposed by Wu et al. [56] with feature genuinization. In the first phase, features obtained from genuine speech were used to train the genuinization transformer. In the second phase, this transformer was converted to enhance the genuine and spoof features’ separation. This transformer was integrated with light-CNN and validated using the ASVspoof 2019 dataset with an EER rate of 4.07%. Li et al. [57] presented a high-frequency feature-based deep CNN model. They extracted long-term variable Q transform (L-VQT) features, and the light-DenseNET model was trained using these features. They validated the model using the ASVSpoof 2019 dataset with various CNN classifiers, including a 0.352% and 3.768% EER on the development and evaluation datasets, respectively.

The literature study reveals that CQCC features and a lateral variant of the CQCC improved the spoofed-speech classification error rate with a statistical or machine learning model to a certain extent compared with other features. High-frequency features with CNN were more prominent in identifying speech with unknown attacks. In CNN, DenseNET, light-CNN, and recurrent neural networks, including RNN, LSTM, and BiLSTM networks, have mainly been used in spoof classification.

## 3. Materials and Methods

### 3.1. Materials

This paper uses ASVSPOOF 2017 dataset provided by [2] for spoofing classification. This dataset focused on replay attacks faced under unseen conditions, i.e., playback devices and replay environments. This dataset is a mixture of both known and unknown scenarios. The audio data were digitized using 16-bit PCM with a 16 kHz sampling rate. Spoofing of audio was created in the wild using different microphones and playback devices in different environments. Creating a dataset from the played spoofing attacks under the uncontrolled setup made it difficult to analyze. This dataset contained replay attacks under different environments. High-quality recording devices can record audio under very low noise conditions, and therefore, they are difficult to identify. The data were divided into three categories: training, development, and evaluation datasets. The speech of the training dataset was captured at a single place, whereas the development set was gathered at two additional sites in addition to the training dataset’s location. A total of 42 speakers participated in dataset creation. Training (train) and development (dev) datasets were developed with 3 and 10 spoofing attacks. The evaluation (eval) dataset recorded audio with 57 types of spoofing attacks. The dataset had a total of 3565 genuine recordings and 14,465 spoofed recordings. Finally, the evaluation, training, and development sets were gathered at two more locations. The summary of the dataset is presented in Table 1 [58].

**Figure 2:**
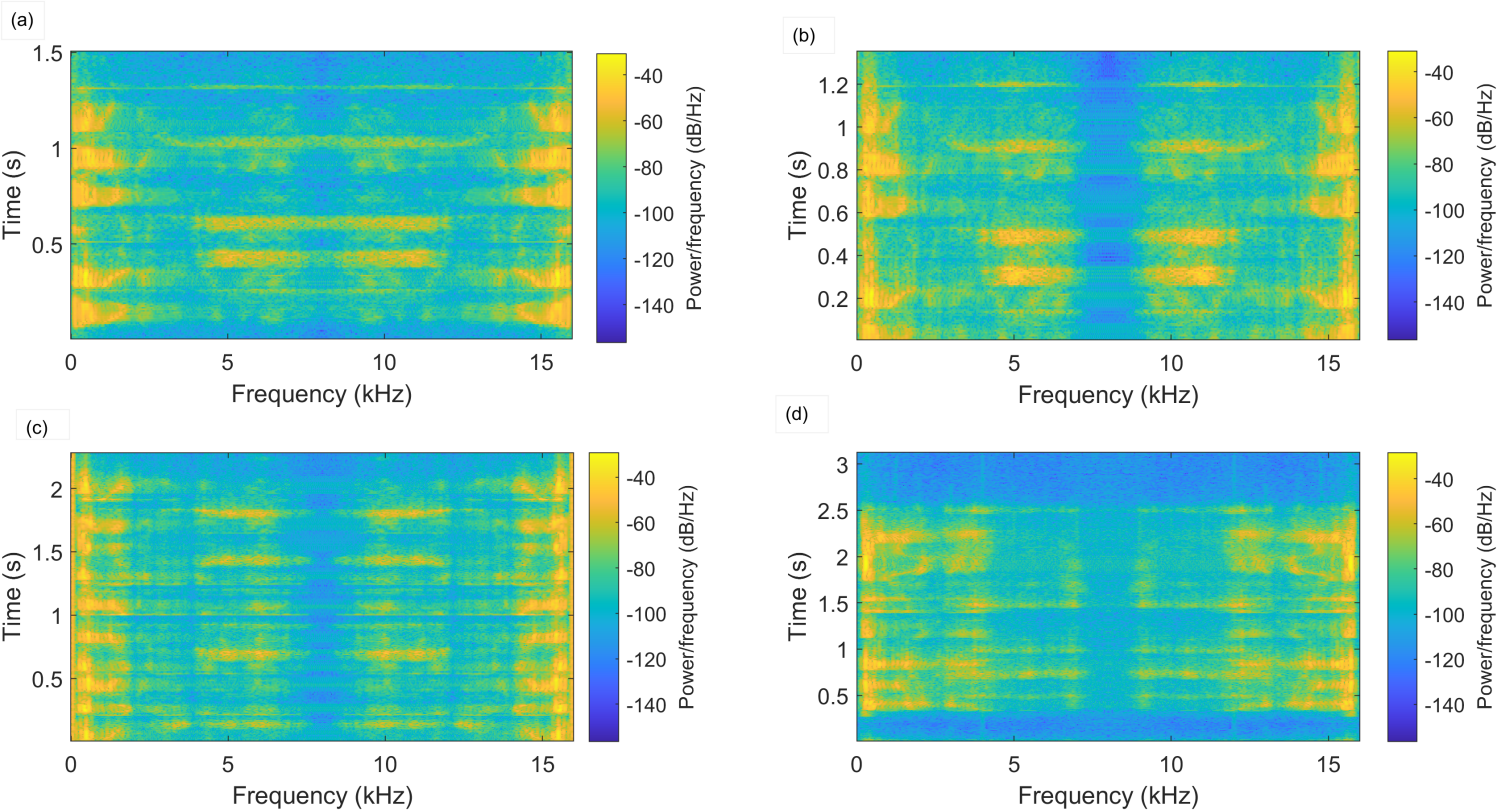
Spectrogram of speech signal under various attacks. (**a**) Genuine speech. (**b**) Spoofed speech using high-quality recording and playback. (**c**) Spoofed speech using high-quality recording and weak-quality playback. (**d**) Spoofed speech using mid-quality recording and low-quality playback.

The spectrum properties of spoofed audio signals for various replay attacks are depicted in Figure 2 using high-/mid-/weak-quality recording devices and playback devices. The spoofing classification for the speech recorded using a high-quality recorder under very low noise conditions (i.e., Figure 2c) was more challenging. The system or model shall overcome the attack reliance in the detection process. The model’s capability highly depends on the sorts of attacks that can be represented by similar patterns and used in the model’s training process. However, it needs previous knowledge of the assault type, which is not a reasonable assumption. Thus, the system must discriminate the spoof audio even if that attack’s data were not utilized for training the model.

### 3.2. Proposed Method

#### Feature Extraction Techniques

Genuine and spoof audio can be discriminated using spectral characteristics. The authors in Reference [17] presented several spectral features to classify genuine audio from spoofed audio. The proposed methods use a combination of several features to train the BiLSTM network. These features are listed below:

Gaussian-Filtered Inverted MFCC (GIMFCC): A replica of the human hearing system was implemented using MFCC computation to imitate the ear’s working principle. Bandpass filters with linear spacing at low frequencies and logarithm spacing at high frequencies were employed in MFCC calculation to keep phonetically crucial aspects of the speech signal. The speech signal consisted of tones, each with varying frequencies. In addition, each tone had a fundamental frequency and Mel-scale-based subjective pitch.

Let $\left\{y{\left[n\right]}_{n=1}^{N}\right\}$ represent a speech frame obtained after framing and hamming windowing. Its power spectrum is calculated using N-point discrete Fourier transform (DFT) as follows:

(2) $$Y\left(k\right)=\sqrt{\left|\sum_{n=1}^{N}y\left[n\right]e^{\left(\frac{-j2\pi kn}{N}\right)}\right|}$$

In the calculation of MFCC, triangle filters spaced uniformly in Mel scales were used [59]. The response of the triangle filter is as follows:

(3) $$H_{i}\left(k\right)=\left\{\begin{array}{cc} 0 & \;k\leq k_{i-1}\\ \frac{k-k_{i-1}}{k_{i}-k_{i-1}} & \;k_{i-1}\leq k\leq k_{i}\\ \frac{k_{i+1}-k}{k_{i+1}-k_{i}} & \;k_{i}\leq k\leq k_{i+1}\\ 0 & \;\mathrm{otherwise} \end{array}\right.$$

where 1<i<Q, *Q* is the number of filters, and $k_{i}$ is the end frequencies of a particular filter. The triangle filters do not consider the correlation between the subband [13]. Gaussian filters providing symmetric and gradually decaying responses can compensate for the possible correlation loss. The Gaussian filters in the same band can be expressed as

(4) $$H{\left(k\right)}_{i}^{GMFCC}=e^{\left(\frac{k-k_{i}}{\sqrt{2}\sigma_{i}}\right)}$$

where $\sigma_{i}=\frac{k_{i+1}-k_{i}}{\alpha }$ is the standard deviation, α controls the variance, and $k_{i}$ is a frequency point representing the mean of ith Gaussian filter.

In calculating conventional MFCC, more emphasis is given to the region of low-frequency signals. However, more promising results can be obtained from the high-frequency regions [11,60]. Therefore, the new filter-bank response was obtained by flipping the original response at the mid-point of the frequency range of the speech signal. The flipped response can be expressed as:

(5) $$\hat{H_{i}}\left(k\right)=H_{Q+1-i}\left(N/2+1-k\right)$$

Power spectrum obtained from Equation (5) is passed through these flipped Gaussian filter banks, referred to as Gaussian-inverted MFCCs (GIMFCCs), and output can be expressed as

(6) $$E^{GIMFCC}\left(i\right)=\sum_{k=1}^{N/2}{\left|Y(k)\right|}^{2}\hat{H_{i}}\left(k\right)$$

Later, the cepstrum coefficients are calculated from the filter bank’s output using Equation (7).

(7) $$\begin{array}{c} C^{GIMFCC}\left(k\right)=\sqrt{\frac{2}{Q}\sum_{i=1}^{Q-1}log\left[E^{GIMFCC}\left(i+1\right)\right]cos\left(\frac{\pi k(i-0.5)}{Q}\right)} \end{array}$$

**Figure 3:**
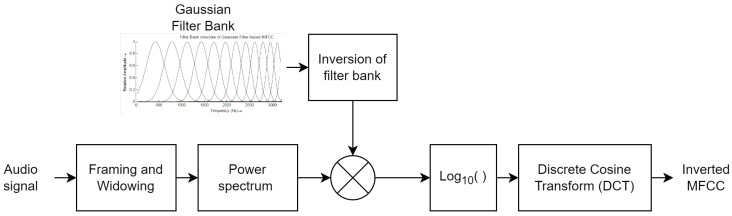
Block diagram representing extraction of Inverted MFCC features emphasizing high-frequency regions.

Dynamic GIMFCC: The above cepstral coefficients contain the information for the given frame and are considered static features. Further information can be obtained by calculating the first (i.e., delta) and second (delta-delta) derivatives of these coefficients, where delta–GIMFCC provides the speech-rate information and delta-delta-GIMFCC provides speech-acceleration information. These delta features can be calculated using Equation (8).

(8) $$\Delta C^{GIMFCC}\left(k\right)=\frac{\sum_{t=-T}^{T}k_{i}C^{GIMFCC}\left(k+i\right)}{\sum_{t=-T}^{T}\left|i\right|}$$

where *T* is the frame number used for coefficient calculation. Thus, these two features are also appended with GIMFCC in the proposed method to improve the performance with negligible computational cost. Figure 3 shows the inverted MFCC feature extraction process.

GTCC and its variant: The human audio response has been modeled using Gammatone filters in many past applications. The Gammatone filter is a function of a sinusoidal tone centered at the $F_{c}$ frequency and Gamma distribution function, which can be written as

(9) $$g\left(t\right)=At^{n-1}e^{-2\pi Bt}cos\left(w_{c}t+\theta \right)$$

where *A* is the amplitude; *n* is the filter order; $w_{c}$ is the central frequency; θ is the phase shift; and *B* represents the filter bandwidth.

The suggested GTCC coefficients are computed similarly as in the MFCC coefficient extraction process, where Gammatone filters have been used instead of triangular filters. The coefficients can be expressed as:

(10) $$GTCC\left(k\right)=\sqrt{\frac{2}{Q}\sum_{i=1}^{Q-1}log\left[E^{GTCC}\left(i+1\right)\right]cos\left(\frac{\pi k(i-0.5)}{Q}\right)}$$

where $E^{GTCC}$ is the power spectrum obtained by passing the speech frame through Gammatone filters g(t). Further features using $\Delta {\left(c\right)}^{GTCC}$ and $\Delta \Delta {\left(c\right)}^{GTCC}$ are also extracted, as explained in the previous subsection.

Spectral Features: Emotion is another influencing factor in the speech signal. It changes the vocal cord vibration, and the speech signal’s spectral features vary. The spectral centroid gives the geometric center of the spectrum. It is a weighted sum of frequency components where weights are assigned based on normalized energy. The spectral flatness determines the degree of periodicity. Spectral entropy accesses the degree of spectral probability’s randomness. It can be analyzed for both voice and unvoiced frames. Thus, it helps to characterize the high-frequency regions. In addition to that, other spectral features, including flatness, skews, a roll of points, and pitch, are also incorporated to improve the classification.

**Figure 4:**
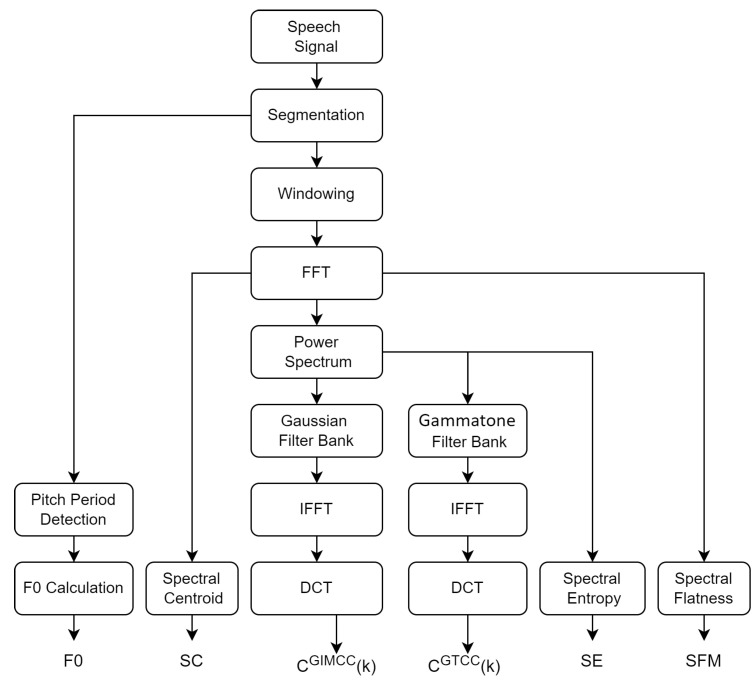
Process flow for feature extraction from the speech signal.

The overall process of extracting various features from the speech signal is shown in Figure 4. These features are used in the proposed optimized BiLSTM network for further classification.

**Figure 5:**
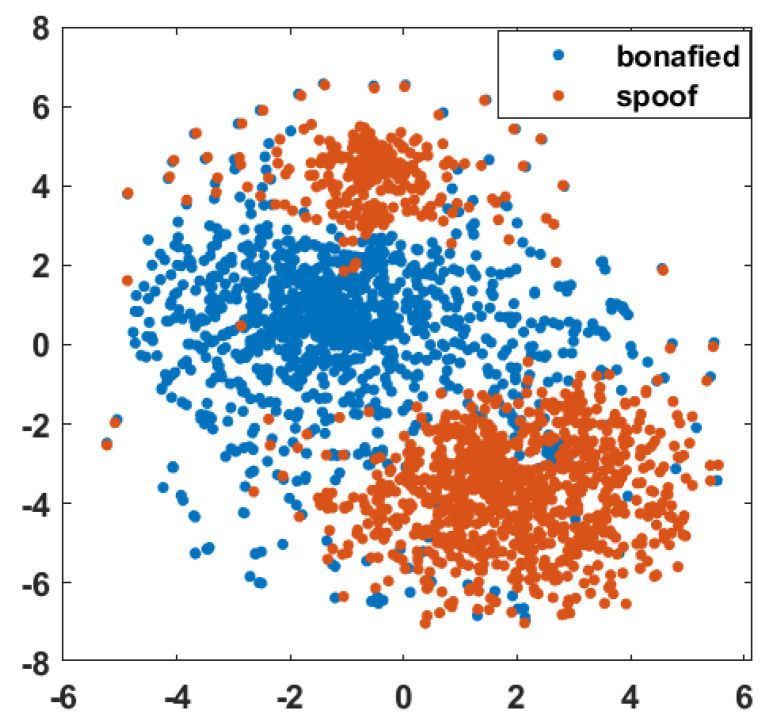
Features’ t-SNE visualization for training dataset.

Before applying the proposed inverted high-frequency MFCC features to construct ASVSpoof systems, their ability to distinguish between authentic and spoof voice segments was studied using the t-stochastic neighborhood embedding (t-SNE) [61] visualization. The t-SNE was plotted from the feature sets of the training dataset. Figure 5 shows that the genuine and spoof class features are separated. Thus, t-SNE provides a significant difference between these two classes.

### 3.3. Proposed Optimized BiLSTM

The recurrent neural network (RNN) is widespread in sequential data processing and classification. The conventional deep network uses the features independently, ignoring the correlation between the present and the following features. In contrast, RNN incorporates the dependencies between the features by storing previous input information at each hidden layer to generate the subsequent output. However, RNN only applies for short periods; LSTM is designed to handle extended periods. A BiSLTM network consisting of two LSTMs is intended to improve the performance further. One LSTM processes input in the forward direction, and the second LSTM processes data in the backward direction. Thus, using data in both forward and backward directions makes the network more efficient than other deep networks. Hence, the proposed method uses a BiLSTM-based deep learning model.

First, let us define the input sequence as $x=x_{1},x_{2},\ldots ,x_{T}$, where *T* is the sequence length. The forward LSTM layer takes this input sequence and produces a hidden state sequence $h_{f}=h_{f1},h_{f2},\ldots ,h_{fT}$, while the backward LSTM layer takes the input sequence in reverse order and produces a hidden state sequence $h_{b}=h_{b1},h_{b2},\ldots ,h_{bT}$.

The hidden state sequence $h_{f}$ was computed using the following equations:

(11) $$\begin{array}{c} i_{t}=sigmoid\left(W_{xi}x_{t}+W_{hi}h_{t-1}+b_{i}\right)\\ f_{t}=sigmoid\left(W_{xf}x_{t}+W_{hf}h_{t-1}+b_{f}\right)\\ o_{t}=sigmoid\left(W_{xo}x_{t}+W_{ho}h_{t-1}+b_{o}\right)\\ g_{t}=tanh\left(W_{xg}x_{t}+W_{hg}h_{t-1}+b_{g}\right)\\ c_{t}=f_{t}\times c_{t-1}+i_{t}\times g_{t}\\ h_{t}=o_{t}\times tanh\left(c_{t}\right) \end{array}$$

where i,f,o, and *g* are the input, forget, output, and cell gates, respectively. $W_{xi},W_{hi},W_{xf}$, $W_{hf},W_{xo},W_{ho},W_{xg}$, and $W_{hg}$ are weight matrices; $b_{i},b_{f},b_{o}$, and $b_{g}$ are bias vectors; and tanh and sigmoid are activation functions.

Similarly, the hidden state sequence $h_{b}$ is computed using the following equations:

(12) $$\begin{array}{c} i_{t}^{\prime }=sigmoid\left(W_{xi}^{\prime }x_{t}^{\prime }+W_{hi}^{\prime }h_{t+1}^{\prime }+b_{i}^{\prime }\right)\\ f_{t}^{\prime }=sigmoid\left(W_{xf}^{\prime }x_{t}^{\prime }+W_{hf}^{\prime }h_{t+1}^{\prime }+b_{f}^{\prime }\right)\\ o_{t}^{\prime }=sigmoid\left(W_{xo}^{\prime }x_{t}^{\prime }+W_{ho}^{\prime }h_{t+1}^{\prime }+b_{o}^{\prime }\right)\\ g_{t}^{\prime }=tanh\left(W_{xg}^{\prime }x_{t}^{\prime }+W_{hg}^{\prime }h_{t+1}^{\prime }+b_{g}^{\prime }\right)\\ c_{t}^{\prime }=f_{t}^{\prime }\times c_{t+1}^{\prime }+i_{t}^{\prime }\times g_{t}^{\prime }\\ h_{t}^{\prime }=o_{t}^{\prime }\times tanh\left(c_{t}^{\prime }\right) \end{array}$$

where $x_{t}^{\prime }=x_{T-t+1}$ and $h_{t}^{\prime }=h_{T-t+1}$ are the inputs to the backward LSTM layer, and the weight matrices and bias vectors have a prime symbol to distinguish them from those of the forward LSTM layer.

Finally, the output of the BiLSTM network was obtained by concatenating the hidden state sequences from both directions:

(13) $$h_{t}^{BiLSTM}=\left[h_{t},h_{t}^{\prime }\right]$$

where [] denotes concatenation.

BiLSTM can capture the long-term dependencies between the data, thus avoiding the vanishing exploding gradient problem. The internal long-term memory helps BiLSTM learn the data explicitly compared with standard deep learning models. However, BiLSTM is more complex, increasing the computational cost due to using two LSTMs. Therefore, an optimum network with few hidden layers and optimum hyper-parameters must be identified before classification.

Deep learning architecture has two model parameters: trainable parameters, i.e., weights adjusted in the training process, and hyper-parameters, i.e., learning rate, hidden layers, neurons in each layer, etc. The selection of these model parameters is critical and plays an essential role in the algorithm’s performance. Previously, grid search algorithm was used to evaluate these parameters. In the grid search method, parameters are divided into even ranges, and the model is assessed for all possible combinations of these parameters; therefore, it is highly time-consuming. Another alternative is the random search, where a random assortment of parameters is evaluated to find the best values. However, it does not guarantee optimum values due to the selection of random combinations.

**Figure 6:**
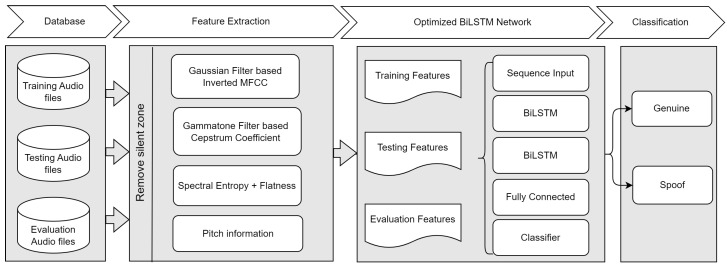
Process flow of the proposed algorithm.

Bayesian optimization is a well-known function to optimize discrete, non-differentiable, and time-consuming algorithms. It is based on the evaluation of the objective function, where the objective function is modeled using the Gaussian process. It employs the Gaussian probabilistic approach to predict the performance and maximize the efficiency in the following samples [62]. Thus, it can find the best parameters in minimal time, in contrast to grid search and random search. In addition, grid and random searches do not have any learning from the accessed set of parameters in the tuning process [63]. Therefore, Bayesian optimization is used in the proposed method to find the best architecture and corresponding parameters for the BiLSTM network. The overall process for spoof audio classification with an optimized BiLSTM network is shown in Figure 6. The dataset is split into training, testing, and evaluation datasets. The training dataset trains the model (optimal BiLSTM). The model’s parameters are changed in accordance with the findings of the comparison and the particular learning. The results of the observations in a second dataset, known as the test dataset, are successively predicted using the fitted model. When the model’s hyper-parameters are adjusted, the test dataset evaluates a model fit on the training dataset. Ultimately, a final model is objectively assessed using the evaluation dataset. Various features, including MFCC and its variant, GTCC and its variant, Spectral features, and Pitch information, were used to train the optimized BiLSTM network.

Optimization of BiLSTM using Gaussian-oriented Bayesian approach: A Bayesian model can find the optimum values of all parameters in minimal time. The process of optimization is categorized into three steps:

Step 1: The first step is to build the Gaussian model for predicting the results of the feature adjustment of the parameters. The hyper-parameters are completely unknown initially; the Gaussian model can realize the scale of setting the hyper-parameters. This scale helps to figure out how much deviation is needed to have drastically different results. For each prediction, the scale generates the Gaussian-distributed curve. The sharpness and variance of this curve suggest how the prediction is close to the consistent training process. The Gaussian curve, which is sharp with smaller variance, provides better consistency in the prediction and optimum tuning of the parameters. Let f(x) be a model function over the known values of training data *x* and $F(x^{*})$ be the model function of a set of test data $x^{*}$. Then, the multivariant Gaussian distribution function can be expressed as

(14) $$\left[\begin{array}{c} f(x_{1})\\ f(x^{*}) \end{array}\right]∼N\left(\left[\begin{array}{c} \mu (x_{1})\\ \mu (x^{*}) \end{array}\right]\left[\begin{array}{cc} \gamma (x^{*},x^{*}) & \gamma (x^{*},x)\\ \gamma (x^{*},x) & \gamma (x,x) \end{array}\right]\right)$$

where μ(x) is the mean and γ(x,x) is the covariance of the training data. $\mu (x^{*})$ and $\gamma (x^{*},x^{*})$ are the mean and covariance of the test data.

This calculation can be extended for a large set of datasets or to multiple dimensions as follows:

(15) $$\left[\begin{array}{c} f(x_{1})\\ \vdots \\ f(x_{\ell }) \end{array}\right]∼N\left(\left[\begin{array}{c} \mu (x_{1})\\ \vdots \\ \mu (x_{\ell }) \end{array}\right]\left[\begin{array}{ccc} \gamma (x_{1},x_{1}) & \cdots & \gamma (x_{1},x_{\ell })\\ \vdots & \ddots & \vdots \\ \gamma (x_{\ell },x_{1}) & \cdots & \gamma (x_{\ell },x_{\ell }) \end{array}\right]\right)$$

The posterior probability of the ℓth point is calculated using previously obtained ℓ−1 observation points as follows:

(16) $$\mu_{\ell }=\sum \left(x_{\ell },x_{1:\ell -1}\right)\sum {\left(x_{1:\ell -1},x_{1:\ell -1}\right)}^{-1}f\left(x_{1:\ell -1}\right)$$

(17) $$\begin{array}{c} \sigma_{\ell }^{2}=\sum \left(x_{\ell },x_{\ell }\right)-\sum \left(x_{\ell },x_{1:\ell -1}\right)\times \sum {\left(x_{1:\ell -1},x_{1:\ell -1}\right)}^{-1}\sum \left(x_{1:\ell -1},x_{\ell }\right) \end{array}$$

The above two equations of mean and variance compute the distribution at any desired point $x_{\ell }$. This creates the Bayesian model for a given objective function.

Step 2: The second step is to find the hyperparameter to maximize the objective function, i.e., accuracy, or to minimize the objective function, i.e., error. In the proposed model, the classification error in the testing dataset was used as an objective function. Therefore, we modeled the error function using the Gaussian process. After the nth iteration, the minimum value of posterior function $f^{*}$ for some test data $x^{*}$ is obtained. After one more iteration, the posterior value of the function is updated, i.e., the updated posterior value of the function is f(x) at point *x*. If maximum iteration has been reached, iteration is stopped here, and the optimum values are calculated by finding $min(f^{*},f\left(x\right))$. Thus, the expected difference between the updated posterior value f(x) and the previous value $f^{*}$ suggests a reduction in the error. This is expressed as

(18) $$\mathrm{Expected}\;\mathrm{improvement}=E\left[f^{*}-min\left(f^{*},f\left(x\right)\right)\right]$$

Equation (18) can be re-written in Equation (19), where *n* indicates the completed iterations, $f^{*}$ gives optimum value obtained through *n* iterations, *p* is the probability density function and *P* is a cumulative density. The objective function is evaluated at a point maximizing an expected improvement.

(19) $$\begin{array}{c} \mathrm{Expected}\;\mathrm{improvement}={\left[f^{*}-\mu_{n}\left(x\right)\right]}^{+}+\sigma_{n}\left(x\right)p\left(\frac{f^{*}-\mu_{n}\left(x\right)}{\sigma_{n}\left(x\right)}\right)\\ -\left[f^{*}-\mu_{n}\left(x\right)\right]P\left(-\frac{f^{*}-\mu_{n}\left(x\right)}{\sigma_{n}\left(x\right)}\right) \end{array}$$

Step 3: All hyper-parameters obtained in all iterations are stored for evaluation. This step needs an evaluation of the objective function using these stored parameters. The least minimum objective function for a specific iteration gives the most optimum values of hyper-parameters of the network.

The overall algorithm to find optimum parameters for the BiLSTM network is as follows (Algorithm 1):
**Algorithm 1** Bayesian optimization algorithm to tune BiLSTM parameters  1:Specify the range of parameters to be optimized  2:Define objective function using network’s validation accuracy and error  3:**while** n≠0**do**  4:    Search hyper-parameter space.  5:    Create BiLSTM network using hyper-parameters.  6:    Train the BiLSTM using the training dataset.  7:    Evaluate the performance of BiLSTM using the Testing dataset.  8:    Score history of BiLSTM.  9:    Update the probability model of BiLSTM.10:**end while**

## 4. Experimentation and Analysis

### 4.1. Experiment Setup

Traditional methods use only a training dataset to train the model. In the proposed model, both training and development datasets are combined and used to train and test the BiLSTM. A total of 3779 (i.e., 80%) audio signals from the training and development datasets were used for training, and the remaining 945 (i.e., 20%) audio signals were used to test the model. The evaluation dataset was used to quantify the accuracy of the proposed model. Traditional statistical methods, e.g., the Gaussian Mixture model, use the logarithm of likelihood ratio to classify the given speech signal as a genuine or spoof type. However, the Equal Error Rate (EER) is the most popular metric to quantify the classifier’s performance in machine learning. EER computation involves false acceptance and rejection, i.e., genuine signals identified as spoofing signals and vice versa. The confusion matrix [64] provides all necessary quantitative parameters to validate the model. The confusion matrix is based on four parameters: true positive (TP), false positive (FP), true negative (TN), and false negative (FN). Where TP gives the correct prediction of the positive class, i.e., genuine audio signals, TN gives the correct prediction of the negative class, i.e., spoof audios, FP gives the incorrect prediction of the positive class, and FN gives the incorrect prediction of the negative class. EER gives an overall accuracy of the classifier and it is a ratio of incorrect predictions (FP + FN) to the total number of the dataset (TP + FP + FN + TN). A lower value of EER presents a better classification accuracy of the classifier.

The first step is the segmentation of the speech signal. A short-time zero-crossing rate is calculated in the ten ms period of a Hamming window. Based on the particular threshold, silent and tail segments are detected with a period of 512 ms. The segment is duplicated for an insufficient length of the segment. Then, features are extracted from each segment and used in the BiLSTM network. During the training step, weights are initialized randomly. The parameters’ range is initialized and supplied to the Bayesian model for optimization. The proposed BiLSTM algorithm was designed in MATLAB V2022, installed on a Pentium I7 processor with 8 GB RAM, a 1.90 GHz PC.

### 4.2. Analysis

The top layer of the BiLSTM is the sequence input layer. We created a BiLSTM structure by placing two BiLSTM layers, each comprising a fully connected layer of 50 neurons, a softmax, and a classification layer. Finding optimum parameters is a challenge in the structuring of neural networks. Therefore, the design incorporated the Bayesian optimization model. The layer information of BiLSTM is shown in Table 2.

**Figure 7:**
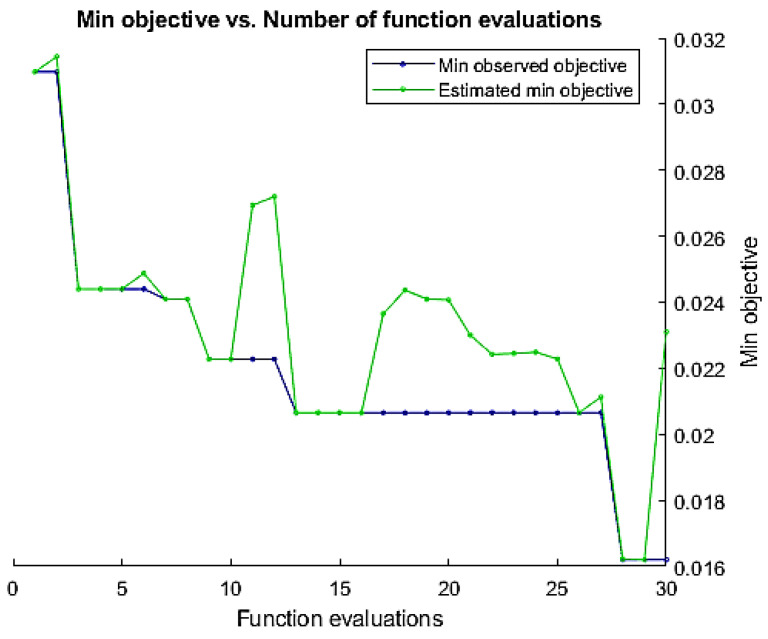
Minimization of the objective function over a number of iterations to find optimum parameters of BiLSTM network.

The Bayesian approach needs a range of parameters to be optimized. The proposed method optimizes the number of hidden layers, learning rate, momentum, and regularization parameters. The validation error minimization was used as an objective function to find the optimum values of these parameters. A total of 400 × 428 features size was used, where 400 is the length and 450 is the feature dimension. At the training time, the weights were randomly initialized. We buffered the feature vectors into sequences of 20 feature vectors with ten overlaps. The mini-batch size was kept at 256. The adaptive learning rate was used, and the drop factor of the learning rate was kept at 0.1. Figure 7 represents the minimization of the objective function based on the tuning of various parameters in the BiLSTM network.

The initial range and optimum value obtained using the approach are presented in the following Table 3. These parameters were used in the BiLSTM structure for classification. After optimization, the number of trainable parameters was only 252,202.

**Figure 8:**
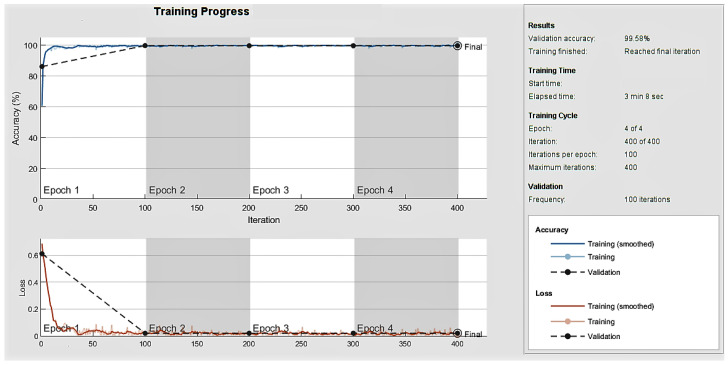
Analysis of accuracy and loss over epochs in the training of optimised BiLSTM network using ASVSpoof2017 dataset.

**Figure 9:**
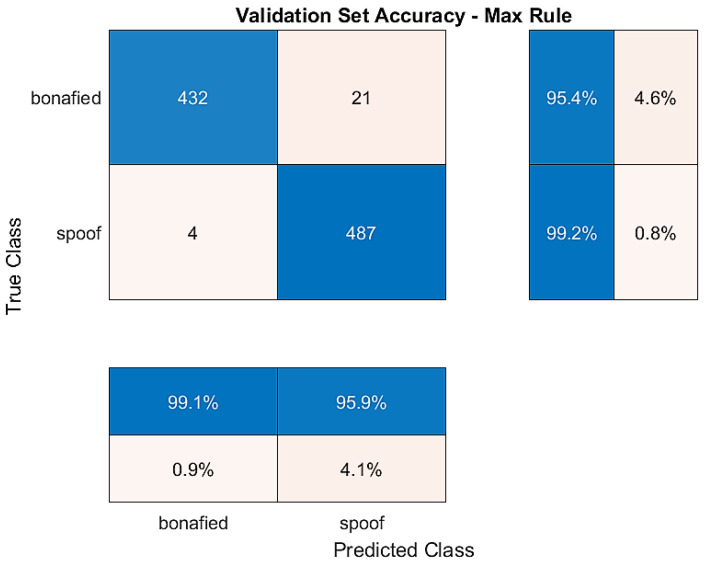
Confusion matrix for validation dataset.

Figure 8 represents the training and validation accuracy obtained over the epoch number, and the confusion matrix for the validation dataset is shown in Figure 9. Diagonal cells show correctly classified observations, and off-diagonal cells show incorrectly classified observations in the confusion matrix. Because of the optimisation, the obtained structure of BiLSTM was minimal, and within only four epochs, the training of the network was fulfilled with a 99.58% validation accuracy and a 2.64% EER.

**Figure 10:**
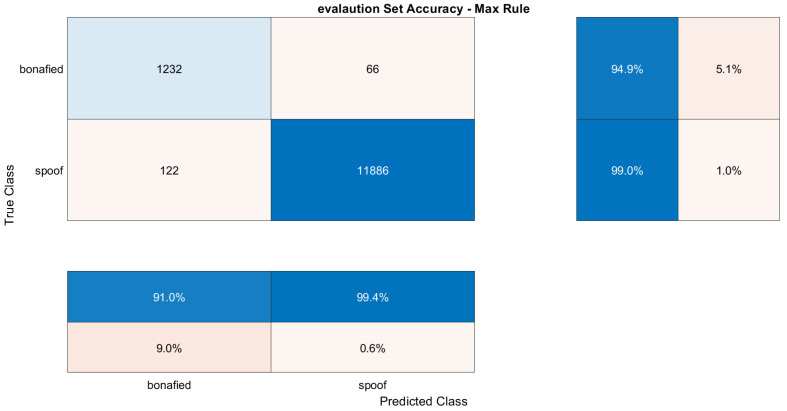
Confusion matrix for evaluation dataset including all attacks.

The training dataset contains genuine and replay attacks based on spoof speech using strong recording devices. The validation dataset contains genuine speech and spoofed speeches, where spoofed speeches are generated using both mid- and high-quality recording devices. The BiLSTM model was trained using both training and development datasets to verify the accuracy against replay attacks. The confusion matrix plotted in Figure 9 and Figure 10 shows the models’ succession to tackle all types of attacks over the validation dataset and evaluation dataset.

The evaluation dataset contains recorded speech using all three (i.e., low, mid, and high) qualities recorded as well as playback devices recording. This makes it challenging. Out of 1298 genuine speeches, 94.9% are correct, and 5.1% are wrong.

**Figure 11:**
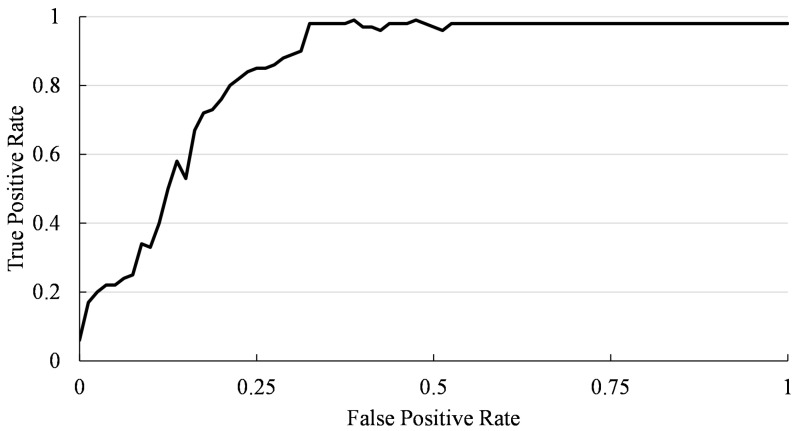
Receiver operating characteristic curve.

The accuracy, precision, and recall analysis for two categorical attacks are listed in Table 4. The experiment received a higher recall rate in comparison to precision. Thus, the proposed model succeeded in finding correct attacks more accurately. The ROC curve obtained by plotting the true positive against the false positive is shown in Figure 11. This curve validates the accuracy of true classification, covering 96% of the area under the curve.

**Figure 12:**
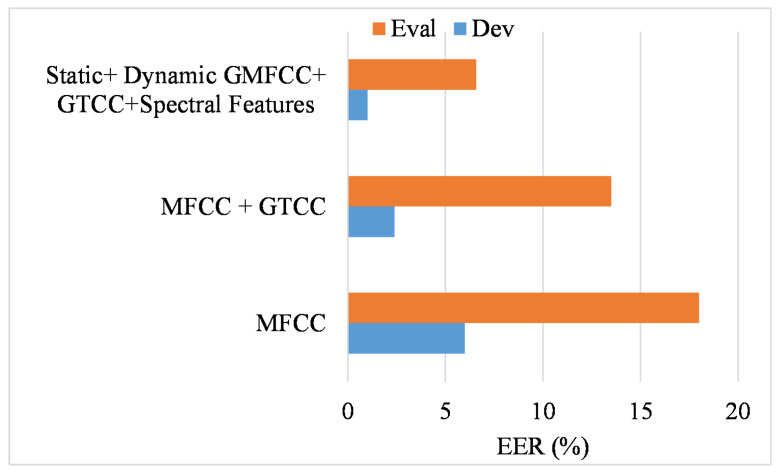
Performance of optimized BILSTM using different feature sets.

In our method, feature integration and BLSTM optimization play an important role. Initially, we explored the impact of the features in the optimized classification model. Three main features were used in the experiment, including MFCC, GTCC, and spectral features. The results of MFCC, the integration of MFCC with GTCC, and the proposed Gaussian-oriented MFCC features sets and their integration with the remaining feature are shown in Figure 12. The MFCC features did not work well; however, integrating MFCC-oriented high-frequency features with GTCC performed well for the development dataset. In contrast, the Gaussian features were better for obtaining a better performance.

Table 5 shows a comparison between the proposed model and other models in the literature. In Reference [65], the authors adopted the conventional CNN-based LCNN-FFT model where the local interpretable model-agnostic explanations (LIME) algorithm was used to find characteristics from the audio signals’ spectral and temporal data. Later, they preprocessed the audio by removing the artifacts from the signals and improved the EER rate of the evaluation dataset to 7.8%. This LIME-based model is computationally complex and needs audio preprocessing. Yoon et al. [66] proposed a new replay attack for the speaker verification system. Initially, they tested various models on the ASVSpoof2017 dataset. They observed that the LSTM classifier using spectrogram features and the RestNET-18 classifier using GD-gram features failed to differentiate the genuine speech from the spoof speech. According to them, the authors of the dataset have not addressed the new replay attacks through their statistical or prototype models.

In Reference [67], a cochlear filter is proposed to obtain cepstral coefficients using instantaneous frequency (CFCCIF). Further, they used quadrature-phase signals using the Hilbert transform with CFCC to improve the feature. They used various CNN models, including light-CNN and ResNet. They obtained a 2.33% EER on the development dataset. However, the EER for the evaluation dataset was 12.88%. One of the weaknesses of this model is that it does not resolve all paradoxes related to instantaneous frequency and its estimation. Bharath and Rajesh Kumar [68] also presented a high-frequency feature-based algorithm. Initially, the glottal excitation spectrum is obtained using adaptive inverse filtering. Finally, CQCC features are extracted from the obtained spectrum for spoofing classification. The EER for the development and evaluation datasets was reduced greatly by the suggested approach to 3.68% and 8.32%, respectively. One of the weaknesses of the CNN is its uncertainty, which may occur due to the use of the softmax layer in the CNN. A Bayesian approach was proposed in Reference [69], which strengthened the assessment of uncertainty in CNN. However, their EER was quite high for a development dataset.

**Table 5:** Comparison of % EER for various features and classifier models using development and evaluation datasets.

Ref	Features	Classifier	Dev EER (%)	Eval EER (%)
[70]	Inverted constant Q-features and CQCC	DNN	2.629	7.777
[67]	CFCCIF and quadrature-phase signals	ResNET	2.33	12.88
[71]	LC-GRNN features from spectrogram	PLDA	3.26	6.08
[72]	MFCC + Fbank	LSTM, GRU RNN	6.32	9.81
[68]	Iterative adaptive inverse filtering -based glottal information + CQCC	Gaussian mixture model	3.68	8.32
[69]	CQCC	LCNN	21.73	8.20
[73]	Normalized log-power magnitude spectrum using Q-transform and FFT	Conventional CNN + RNN	3.95	6.73
[65]	Local interpretable model -Agnostic explanations	LCNN-FFT	7.6	10.6
[66]	Spectrogram	LSTM	-	21.0602
[66]	Group delay-Gram	ResNET-18	-	35.35
[11]	Linear filter bank-based high frequency features	DenseNET + BiLSTM	2.79	6.43
[74]	MFCC + CQCC High-frequency features	DenseNET + LSTM	3.62	8.84
[47]	Improved TEO	LCNN	6.98	13.54
[43]	CQCC + deep learning features	LSTM		7.73
Proposed	Static + dynamic GIMFCC + GTCC+ spectral features	Optimized BiLSTM	1.02	6.58

In Reference [47], TEO features were improved with signal mass consideration. The TEO operator only uses energy in low-frequency regions, and signal mass compensates for the energy of high-frequency regions, providing a more precise signal energy estimation. A lightweight CNN model achieved a 6.98% and 13.54% EER. In contrast, the GMM model performed well, with a 10.75% EER on the evaluation dataset. Chen et al. [72] validated the two recurrent models, i.e., RNN, LSTM, and Gated Recurrent Unit (GRU) network. They evaluated these models using the MFCC and FBank features. The GRU model performed best with FBank features, with an EER of 6.32% and 9.81% for the development and evaluation datasets. In Reference [74], a silent segment was extracted from the speech signal using a zero-crossing rate, and its CQCC high-frequency features in the 3 to 8 kHz frequency range were obtained. In addition, they proposed a DenseNet-LSTM network compared with CNN-RNN as a classifier. This model succeeded in lowering the EER to 3.62% and 8.84% on the development and evaluation datasets, respectively. Later, the authors in Reference [11] modified the classifier to a DenseNet-BiLSTM network with an attention mechanism. Their model outperformed the LSTM and GMM models with an EER of 6.43% on the evaluation dataset. However, in both models, high-frequency features heavily rely on segment-based linear filter banks, which are sensitive to the background sound and playback devices. A convolutional feature using a light convolutional gated RNN (LC-GRNN) was extracted in Reference [71]. Three machine learning models were tested for all possible attacks, including SVM, linear discriminant analysis (LDA), and probabilistic LDA. Among all classifiers, probabilistic LDA performed better. However, the major drawbacks of RNN are its vanishing gradient and long-term dependency. Huang et al. [75] used CQCC features and features obtained using deep learning from the raw data. These two features were used in the LSTM network for classification and achieved a 7.73% EER on the evaluation dataset.

High-frequency features work well, as depicted in Reference [11]. They used a triangle filter as a linear filter bank to obtain high-frequency features, and they used a combination of DenseNET and BiLSTM as a classifier, achieving a 6.43% EER. Due to the prominent characteristics of the Gaussian filter, as explained in Section 1, The Gaussian filter-based high-frequency features with complementary features intuitively worked well with the optimized BiLSTM model. Due to the discriminating high-frequency features of spoofed and genuine speech signals, the BiLSTM model could discriminate speech with the best classification accuracy and lower EER. In addition, BiLSTM overcomes the weakness of RNN, tackling the problem of the vanishing gradient, and, therefore, performed well compared with the RNN. The proposed model succeeded in obtaining an overall 1.02% and 6.58% EER on the validation and evaluation datasets, which were the least among all the models, as presented in Table 5.

## 5. Conclusions

The manipulation of genuine speech is a common threat and a prime concern for the system’s vulnerability. Though baseline features are reliable, the fusion of features and better classifiers is expected to strengthen the system. The present study reveals that high-frequency features can easily differentiate spoof speech from genuine speech. In addition, the complementary features based on the high-frequency region of speech in the signal can help to improve the classification accuracy. This work presents the use of high-frequency features for spoof-speech detection. A Gaussian filter offering good correlation among the subband and allowing a smooth transition between the subbands is proposed to extract high-frequency features in contrast to linear or triangular filter banks. Inverted MFCC-based high-frequency features using a Gaussian filter are obtained from the high-frequency region of speech signals. To use these features efficiently, BiLSTM-based CNN architecture offering a correlation between the past and present features is explored. Further, optimizing the BiLSTM network using the Bayesian algorithm showed a minimal architecture with the best hyper-parameters. The integrated features with the optimized BiLSTM network performed better than base and state-of-art algorithms.

The proposed model was evaluated using the ASVSPoof 2017 dataset. In the base algorithm of GMM, the training dataset was used in training the algorithm. In contrast, both the training and development datasets were used in the training process of the network, and performance was evaluated for both development and evaluation datasets in the proposed approach. The proposed model achieved a 1.02% and 6.58% EER with a relative reduction of 78%. The state-of-the-art comparison with other CNN classifier-based algorithms states that the proposed model achieved better performance with minimal architecture.

The limitation of our work is that we did not investigate the performance of our method in cross-dataset scenarios. Future research will enhance the anti-spoofing countermeasures’ capacity to generalize under many circumstances. In addition, the effect of noise is more considerable at high frequencies because it contains information, so any small noise significantly impacts the signal [11]. Although the experimental results show the efficiency of the proposed features, these features may need to perform better for noisy signals. Features describing the glottal source are another alternative MFCC, which can be calculated using single-frequency filtering, zero-time windowing, and zero-frequency filtering. Therefore, further investigation in the presence of noise, as well as adopting alternate features, is required.

## Figures and Tables

**Table 1:** ASVSpoof2017 dataset summary and its utilization in the experiment.

	Dataset Summary	Use of Dataset in the Experiment
Speech format	Precision: 16 Bit PCM, Sampling rate = 16 kH	Precision: 16 Bit PCM, Sampling rate = 16 kHz
Spoofing types in train/dev/eval	3/10/57	3 /10/ 57
Total speakers in train/dev/eval	10/8/24	18 from train, 24 from eval
No. of genuine speech in train/dev/eval	1507/760/1298	2267 from train and dev to train the network, 1298 from eval dataset
No. of spoofed speech in train/dev/eval	1507/950/12,008	2457 from train and dev to train the network, 12,008 from eval dataset

**Table 2:** Proposed BiLSTM network’s parameters.

Layer	Layer’s Name	Main Parameters	Other Parameters
1	Sequence Layer	Size of training features	-
2	BiLSTM	50	Returned Sequences = True
3	BiLSTM	50	Returned Sequences = True
4	Fully Connected Layer	-	-
5	Dense	-	Activation softmax
6	Classification Layer	2	-

**Table 3:** Range of hyper-parameters initialized and obtained optimum value.

Hyper-Parameter	Range	Optimum Value
Section Depth	2 to 6	2
Learning Rate	10${}^{-6}$, 10${}^{-5}$, 10${}^{-4}$, 10${}^{-3}$, 10${}^{-2}$, 10${}^{-1}$, 1	10${}^{-3}$
Momentum	0.2, 0.3, 0.4, 0.5, 0.6, 0.8, 0.90, 0.98	0.5
L2-Regularization	1 × 10${}^{-10}$ to 1 × 10${}^{-2}$	1 × 10${}^{-4}$

**Table 4:** Accuracy, precision and recall analysis for development and evaluation datasets.

Dataset/Attack Types	Accuracy	Precision	Recall
Validation dataset	97.35	95.78	99.08
Evaluation dataset	98.58	94.92	90.98

## Data Availability

The authors confirm that the data supporting the findings of this study are available within the article [2].

## Abbreviations

The following abbreviations are used in this manuscript:

MFCC	Mel Frequency Cepstral Coefficients
GIMFCC	Gaussian-Inverted MFCC
GTCC	Gammatone Cepstrum Coefficient
LPC	Linear Prediction Coefficients
LFCC	Linear Frequency Cepstrum Coefficients
ECG	Electrocardigram
CQCC	Constant Q Cepstral Coefficients
CQT	Constant Q Transform
GMM	Gaussain Mixture Model
CNN	Convolutional Neural Network
RNN	Recurrent Neural Network
LSTM	Long short-term memory
BiLSTM	A bidirectional LSTM
SVM	Support Vector Machine
EER	Equal Error Rate
XGBoost	Extended Gradient Boost
ReLU	Rectified Linear Unit
ResNET	Residual Neural Network
PCM	Pulse Code Modulation
t-SNE	T-Stochastic Neighborhood Embedding
